# Fibromatosis of the hand associated with EMO syndrome: A Case report

**DOI:** 10.1186/1471-5945-4-17

**Published:** 2004-11-08

**Authors:** Carolin Appelhans, Frank Breuckmann, Andreas Bastian, Peter Altmeyer, Alexander Kreuter

**Affiliations:** 1 Dept. of Dermatology, Ruhr-University Bochum, Gudrunstr. 56, D-44791 Bochum, Germany; 2 Dept. of Internal Medicine, St. Josef Hospital Bochum, Gudrunstr. 56, D-44791 Bochum, Germany

## Abstract

**Background:**

EMO syndrome, defined as a triad including exophthalmus, pretibial myxedema and osteoarthropathia, is a rare condition in patients suffering from hyperthyreosis.

**Case presentation:**

We here describe an interesting case of EMO syndrome associated with unilateral fibromatosis of the hand and an initial stage of generalized myxedema of the skin. To our knowledge a similar case has not yet been described in literature though reports about associated fibromatosis, e.g. located retroperitoneally, already exist. Familiar explanations include its initiation by autoimmune processes or aberrant T-cell cytokine stimulation leading to an overwhelming production of glycosaminoglycans.

**Conclusion:**

Interpreting our case in context with previous reports we conclude that associated fibromatosis induced by autoimmune processes may affect a variety of different localizations and therefore requires careful monitoring. A therapeutical attempt by using UVA1 irridation for pretibial myxedema remained without a satisfying regression.

## Background

EMO syndrome is a rare condition seen in patients suffering from hyperthyreosis. It is defined as a triad of exophthalmus, pretibial myxedema and osteoarthropathia occurring in less than 1% of patients suffering from Graves' disease [1]. We here describe an unusual case of EMO syndrome associated with unilateral fibromatosis and an initial stage of generalized myxedema of the skin.

## Case presentation

**Figure 1:**
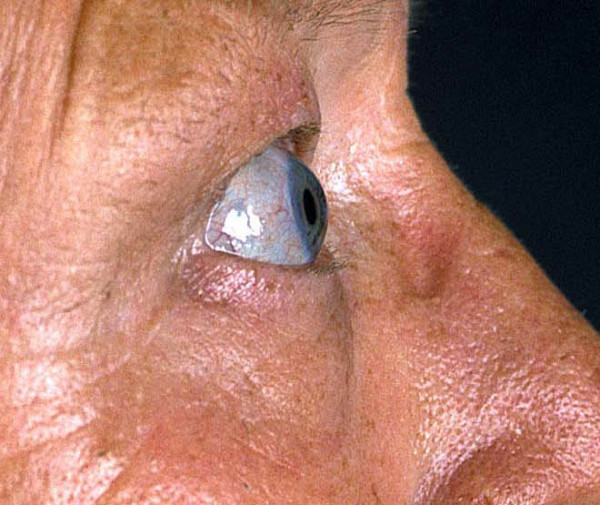
Pretherapeutic clinical appearance of a 64-year-old patient consistent with the diagnosis of EMO syndrome, exophthalmus (a), combined lymphatic congestion and pretibial myxedema (b) and palmoulnar fibromatosis (intrasurgical, c).

In October 2003, a 64-year-old male Caucasian patient was admitted with aggravating pretibial myxedema. Five years ago, he was diagnosed with hyperthyreosis caused by Plummer's disease. Laboratory findings revealed a repressed TSH of 0.01 mU/L (0.4–4.0 mU/L). The patient was treated with radioiodine therapy. Today he suffers from hypothyreosis and a daily substitution of 100 μg L-Thyroxin is performed. Within the months following the radioiodine therapy, an erythema progressing into a manifest pretibial myxedema developed, followed by exophthalmus and fibromatosis of the right hand within the next three years. Surgical orbita decompression due to massive exophthalmus was performed, followed by a subsequent correction of the ocular muscles. Additionally, the fibromatosis of the right hand was excised. Retrospective histological findings revealed a regressive sclerotic fibrosis of firm kollagenous fibers embedded in soft and fat tissue were consistent with a generalized fibrosing process as known in myxedema. Within the last months, decent mucous plaques of the upper limps decreased. The patient now reported progressing congestion of lymph and decreasing flexibility of the lower legs caused by the pretibial myxedema. Clinical examination confirmed massive pretibial myxedema, lymphatic congestion of the lower legs, generalized myxedema accentuating the upper limps, a residual postsurgical nodular tumor of the ulnar aspect of the right hand, exophthalmus, and severe hypertrophic osteopathy of the distal phalanges with clubbed fingers and hippocratic nails (Fig. 1). A Complete check-up including autoantibodies remained unremarkable. Under substitution of 100 μg L-Throxin daily we found the following thyroidal values: T3 0.94 ng/ml (0.59–1.74), T4 10.17 μg/dl (4.50–12.00), TSH 0.47 μIE/ml (0.38–4.70), fT3 2.64 pg/ml (1.45–3.48), fT4 1.44 ng/dl (0.71–1.85). There were no hints for additional fibromatosis. The classical combination of exophthalmus, pretibial myxedema and acropachy led to the diagnosis of EMO syndrome associated with fibromatosis of the right hand and a beginning generalized myxedema of the skin.

## Conclusions

Grave's disease is known to eventually develop after radioiodine therapy [2] and this may be followed by a EMO syndrome. To our knowledge this is the first report about a EMO syndrome combined with coexistent palmoulnar fibromatosis. The patient was treated with lymphdrainage and physiological compressive therapy. As Farr et al. [3] reported the successful use of PUVA in a case of scleromyxedema, we initiated a therapeutic attempt with conventional UVA1 irradiation five times a week for one week followed by three times per week for three consecutive weeks (20 J/cm^2 ^single dose, 280 J/cm^2 ^cumulative dose). While the congestion of lymph clearly improved, pretibial myxedema remained without any signs of regression.

A massive recurrence after excision of thyroid dermopathy, as described in literature, could not be observed in our patient during a follow-up of 2.5 years.

Generalized fibrosing processes are thought to be based on an accumulation of glycosaminogylcans. Common explanations comprise its initiation by autoimmune processes, e.g. thyreotropin receptors on fibroblasts, or aberrant T-cell cytokine stimulation, leading to overwhelming glycosaminoglycan production [4] Fibrosing processes such as retroperitoneal or sellar associated with EMO syndrome or multifocal fibrosclerosis, respectively, have been previously reported [5,6]. Nevertheless, coexistent palmar fibromatosis so far has not been described.

Therefore, we conclude that concomitant fibromatosis might appear in various localizations requiring elaborated diagnostic procedures and monitoring in all patients affected. We started low-dose UVA1 irradiation of the pretibial myxedema known to be able to degrade pathologic collagenous architecture by inducing dermal matrix-metalloproteinases as well as by decreasing abnormal cytokine liberation following T-cell apoptosis [7]. Nevertheless, our patient discontinued UVA1 phototherapy due to non response after a cumulative dosage of 280 J/cm^2 ^UVA1 (seven treatment sessions). However, we suggest that phototherapy might also be considered as an adjunct therapeutic alternative in persistent EMO syndrome.

## Competing interests

The author(s) declare that they have no competing interests.

## Authors' contributions

C.A. attended to the patient, participated in the design of the case report, and drafted the manuscript. A.K. conceived the study. F.B., A.B. and P.A. participated in its design and coordination.

All authors read and approved the final manuscript.

## Pre-publication history

The pre-publication history for this paper can be accessed here:



## References

[B1] Goette DK (1980). Thyroid acropachy. Arch Dermatol.

[B2] Niepomniszcze H, Pitoia F, Goodall C, Manavela M, Bruno OD (2001). Development of Graves' hyperthyroidism after radioiodine treatment for a toxic nodule: is the hyperthyroidism always triggered by 1311 therapy?. Thyroid.

[B3] Farr PM, Ive FA (1984). PUVA treatment of scleromyxoedema. Br J Dermatol.

[B4] Chang TC, Wu SL, Hsiao YL, Kuo ST, Chien LF, Kuo YF, Change CC, Chang TJ (1994). TSH and TSH receptor antibody-binding sites in fibroblasts of pretibial myxedema are related to the extracellular domain of entire TSH receptor. Clin Immunol Immunopathol.

[B5] Armigliato M, Paolini R, Bianchini E, Monesi G, Zamboni S, D'Andrea E (2002). Hashimoto's thyroiditis and Graves' disease associated with retroperitoneal fibrosis. Thyroid.

[B6] van der Pol R, Nieuwenhuis MG, Mourits MP (1999). Multifocal fibrosclerosis presenting as Grave's orbitopathy. Bilateral exophthalmos associated with retroperitoneal and sellar fibrosis. Graefes Arch Clin Exp Ophthalmol.

[B7] Dawe RS (2003). Ultraviolet A1 phototherapy. Br J Dermatol.

